# Supplementary material for: Are carbon emissions trading and green financial instruments synergistic? -Comprehensive quantitative research based on content analysis
Source: PLoS One. 2024 Mar 7;19(3):e0298601. doi: 10.1371/journal.pone.0298601 (PMC10919601; doi:10.1371/journal.pone.0298601)
Supplement: S1 Data — (ZIP) [file pone.0298601.s001.zip › Data/地级市Z_F工作报告.zip!/┴╔─■/╔≥╤⌠╩╨/└┤╫╘╣1⁄2╓┌║┼í╛┬φ┐╦╩2╛▌═°í┐_2019.docx]

各位代表：

　　现在，我代表市政府向大会报告工作，请予审议，并请市政协委员提出意见。

　　一、2018年工作回顾

　　过去一年，是全面贯彻党的十九大精神开局之年。在市委正确领导下，我们以习近平新时代中国特色社会主义思想为指导，认真贯彻落实习近平总书记在辽宁考察时和在深入推进东北振兴座谈会上的重要讲话精神，按照高质量发展要求，坚持稳中求进工作总基调，坚持以供给侧结构性改革为主线，积极推进“一带五基地”建设，深入实施辽宁“五大区域发展战略”，紧紧依靠全市人民，攻坚克难，大干实干，推动沈阳振兴发展迈出坚实步伐。

　　经济运行稳中有进。预计地区生产总值增长5%左右，固定资产投资增长15%，社会消费品零售总额增长8.3%，进出口总额增长16.7%，增速均高于上年。质量效益稳步提高。一般公共预算收入720.6亿元，增长10%；城镇居民人均可支配收入44170元、增长6.8%，农村居民人均可支配收入16620元、增长7.5%；1-11月规模以上工业企业利润总额增长7%；万元地区生产总值能耗下降3.6%。发展活力持续增强。新增市场主体17.3万户，民间投资增长18%，创新创业日趋活跃；新引进各类人才1万余人，吸收高校毕业生11万人，人才保持回流趋势。城乡环境明显改观。三城联创取得突破性进展，基础设施不断完善，一些受“邻避效应”困扰的公用设施建设问题得到解决，空气质量优良天数增加29天，建成区黑臭水体全面消除。人民生活不断改善。财政用于重点民生支出达到776.4亿元、增长7.8%，基本民生保障标准稳步提高，教育、医疗、住房、养老等服务保障能力持续提升，人民群众从振兴发展中获得更多实惠。

　　一年来，我们主要做了以下工作：

　　(一)深入推进营商环境建设。坚持问题导向、标本兼治，8个案例入选中国营商环境优化最佳实践案例，企业和群众对营商环境认可度持续提高。

　　专项整治取得新成效。开展政务窗口服务质量差、乱检查乱收费乱罚款、垄断性行业服务、政府和企业失信、中介服务不规范、政策落实“最后一公里”六大专项整治，解决各类问题118批次969个，政务窗口服务持续改进，涉企收费普遍实行清单管理，企业办水、办电、办气等更加便利，偿还拖欠工程款年度目标如期实现，红中介、灰中介得到全面清理，发布惠企政策清单432项。开展办事难问题整治，推出领办代办、减证便民、延时错时服务等一系列举措，38项整治任务有效落实。

　　放管服改革不断深化。开通商事主体综合服务平台，推行一证零表、证照联办，企业开办时间压缩到3.5天。完善“多规合一”项目管理平台，强化前期业务协同，开展区域评估，实施规划联审、施工图联审、多测合一和联合验收，工程项目审批时限压缩一半以上。运行综合便民服务平台，制定185个热点需求侧索引化办事指南，出入境、身份证等业务实现24小时自助服务。建设一体化在线政务服务平台，启动“一网一门一次”改革，70%的政务服务事项实现“网上办”。

　　要素条件持续改善。落实税收新政减税42.7亿元，取消9项行政事业性收费，推行工伤保险浮动费率政策。降低企业运营成本，一般工商业电价以及供水、燃气配套费下调10%以上，货运车辆“三检合一”全面实施。着力缓解企业融资难问题，应急转贷资金周转投放260亿元，启动助保贷业务，新增债券融资85亿元，新增贷款规模增长13.3%。

　　(二)加快推动新旧动能转换。着力扩增量、优存量、抓融合、补短板，新的发展动能持续集聚成长。

　　项目建设成效显著。积极走出去、请进来，扩大招商引资，强化项目服务，破解受阻难题，全市上下抓项目、争项目意识不断强化，项目建设数量在增多，项目建设质量在提升。全年开复工亿元以上项目1048个，同比增加422个。作为国家7个重大利用外资项目之一、投资30亿欧元的华晨宝马新工厂开工奠基。华晨雷诺整车、新松机器人未来城、恒大文化旅游城等43个50亿元以上项目落地开工，重大产业项目建设取得突破性进展。全年实际利用外资增长41%，引进内资增长15%。

　　制造业加快转型升级。实施高端装备创新工程，32个04专项、8个02专项结题验收，120万吨乙烯三机达到国际先进水平，±800千伏高端换流变压器获得国家科学技术进步最高奖，大型风洞用压缩机组等重大技术装备研制取得突破，高端装备制造业产值占比达到23.1%。大力培育战略性新兴产业，拓荆、芯源、富创、中科仪等IC装备产业化项目集中实施，新能源汽车产量从2100辆提高到2.3万辆，机器人、航空、生物医药等产业产值分别增长11%、14%、32%。支持企业管理升级，启动5G应用示范工程，华为云、紫光云、国信优易云等云平台上线运行，沈鼓、富创精益管理道场投入使用，参训企业生产效率明显提升。

　　现代服务业加快发展。新增金融及金融服务机构36家，融盛财险、渤海银行沈阳分行开业运营，成大生物获批赴港上市。申通快递东北总部基地和居然之家、唯品会等企业的东北分拨中心落户沈阳，京东“亚洲一号”智慧物流中心、空港口岸物流快件监管中心加快建设，快递业务量增长25.2%。浑南、于洪等4座电商产业园集聚企业660余家，限额以上企业网上商品零售额增长33.4%。盛京皇城创建国家5A级旅游景区取得积极进展，“浪花爱上雪”“梦回大辽季”等旅游品牌影响力持续扩大，旅游总收入增长15%。莫子山国际雕塑主题公园落成开放，玖伍文化城等文化综合体加快发展，文化及相关产业产值增长18.9%。举办APEC中小企业技展会等活动406项，交易额突破3000亿元。

　　乡村振兴实现良好开局。扎实推进农业供给侧结构性改革，保障农产品安全供给，调减玉米种植面积16万亩，建设高标准农田20.9万亩、设施农业1.3万亩，农业综合机械化率达到85%。大力推行“公司+基地+农户”模式，带动40万农户参与产业化运营，家庭农场超过1万家，成立全省首批农村集体股份合作社。推进一二三产融合发展，开工建设农产品加工项目22个、休闲农业项目62个，农村电商主体增长1.7倍、网上交易额增长1.2倍，沈阳西芹、沈阳大米等农业品牌加速走向全国市场。建设美丽宜居示范村12个，建成“四好农村路”1400公里。县域经济呈现积极变化，一般公共预算收入、规模以上工业增加值、固定资产投资分别增长15.6%、10.2%和18.4%。

　　军民融合创新扎实开展。围绕军转民、民参军和军民合作，实施军民融合“十个一”专项行动计划，出台支持军民融合产业发展16条政策措施。浑南、沈北、皇姑、法库等军民融合产业园区加快建设，沈飞A220系列新工厂、国产燃气轮机产业基地等59个项目扎实推进，军民融合产业联盟单位达到150家，军民融合产业产值超过700亿元。

　　(三)全面深化改革扩大开放。统筹推进各项国家级试点示范，改革全面发力、多点突破，开放平台通道加快建设。

　　全面创新改革试验取得显著成效。如期完成制造业创新中心建设等27项国务院授权的试点任务，沈阳化工大学“三定向”订单式科技创新和成果转化机制、中航发黎明公司军品竞争性采购制度2条经验在全国推广，机床集团市场化债转股、中德产业园承诺制审批等12项成果入选全国创新改革试验百佳案例。

　　国资国企改革迈出重要步伐。出台4批共109条政策清单。“一企一策”改革不断深化，机床集团入选国务院国企改革“双百行动”，东药集团引入民营资本大股东，北方重工集团进入司法重整程序，沈鼓集团等企业改革取得新成果。混合所有制改革提速推进，混改率从上年的23%提高到54.8%。“3+1+N”国资平台有序运营，国有资本流动性逐步增强，工商、金融、文化等各领域国有资产实现集中统一监管。化解历史包袱进程加快，139个中央和省直企业“三供一业”项目全部分离移交，245户“僵尸企业”处置提前完成，427户厂办大集体改革启动实施。

　　重点专项改革全面展开。市级政府机构改革顺利完成，事业单位大幅精简整合。建立健全开发区规划体系和绩效考核体系，推行“管委会+平台公司”运营模式，沈阳高新区在全国排名由43位上升到39位，沈阳经开区由49位上升到21位，辉山经开区由164位上升到134位。深化投融资体制改革，完善政府性基金运营体系，设立母基金及PPP基金、产业引导基金和国企改革基金，实施多层次资本市场建设三年行动计划。推进县乡财政体制改革，出台财政增量返还政策，乡镇全部恢复或设立国库。整合城市管理和执法职能，完成城区环卫扫保市场化改革。

　　民营经济稳步发展。开展民营经济大调研，制定进一步促进民营经济发展若干意见，全面启动“万人进万企”主题活动。全年实现“个转企”2114户、“小升规”116户，禾丰牧业再度入选中国民营企业500强，新增292家成长性中小企业在辽股交挂牌，3家企业新三板挂牌，风景园林已完成辅导备案，麟龙股份已申报国家证监会。民营企业贡献了全市40%以上的地区生产总值、50%以上的税收、60%以上的技术创新成果、80%以上的城镇就业，成为振兴发展的中坚力量。

　　开放合作取得新成果。加快自贸区沈阳片区建设，93项“证照分离”改革事项全部落地实施，集报集缴通关等26项举措全省推广，国际贸易“单一窗口”覆盖率超过80%，沈阳综保区桃仙园区通过国家验收。完善中德产业园“一个平台、五大体系”，80个项目签约落地。复航沈阳—法兰克福航线，开通沈阳—洛杉矶航线。跨境电商综合试验区启动建设，跨境人民币结算业务拓展到86个国家和地区。新批“一带一路”投资项目12个，新松机器人、何氏眼科等企业加快海外并购。世界冬季城市市长会议等重大活动在沈举办，新增巴基斯坦卡拉奇等3座友好合作关系城市。联东U谷国际汽车研创园等218个京沈合作项目顺利实施，沈阳经济区物流、旅游、会展等一体化取得新发展。

　　(四)着力激发创新发展活力。加快自主创新示范区建设，不断完善创新体系，多主体联动、全方位推进的创新发展局面加快形成。

　　技术创新能力明显增强。持续抓好“1123”工程，承担国家重大科技项目911项，解决关键共性技术769项，研发新产品、新工艺522项，获得国家科技奖励9项。新松机器人在平昌冬奥会闭幕式“北京8分钟”惊艳世界，中科院沈阳自动化所“海星6000”创造了我国有缆遥控水下机器人深潜纪录。全市科技型中小企业达到1431家，高新技术企业达到1230家，涌现出8家“瞪羚”企业和1家“独角兽”企业，初步形成了创新型企业梯队成长格局。

　　重大创新平台加速布局。国家机器人创新中心揭牌成立，材料科学国家研究中心开工奠基，中国科学院大学机器人学院、中国科技大学材料学院、国家眼基因库加快建设，中科院机器人与智能制造创新研究院投入使用。全市新型研发机构达到8家，国际合作研发机构达到58家。以“先进材料+智能制造”为特色的创新平台体系日益完善。

　　协同创新水平加快提升。实行市长与大学校长、科研院所长联席会议制度，组建沈阳科技创新大联盟，产业创新联盟达到50家。完善科技成果转化服务体系，常态化举办科技成果转化对接活动，培育技术转移示范机构41家，培养技术经纪人200余名。全市技术合同成交额增长15.9%，本地转化占比提高到28%，高新技术产品产值占规模以上工业比重达到55.4%。

　　创新创业环境进一步改善。中国(沈阳)知识产权保护中心启动建设，全市申请专利数量增长20%、专利授权增长32.3%。坚持引才育才留才多措并举，制定59项人才政策实施细则，认定高层次人才2880人，“三引三回”活动储备人才3700人，评选表彰首批25名“盛京大工匠”。推进国家双创示范基地建设，开展双创活动1600余场，引入中关村领创空间、北京双创街等双创资源，市级以上众创空间、孵化器、加速器达到124家，集聚创业人员10万余人。

　　(五)不断提升城市承载能力。坚持规划统筹、统筹规划，大力推进各类城市病问题治理，城市功能更加完善。

　　积极促进城市集约发展。划定全域“三区三线”，出台城市停车、绿地系统、变电站站址路径等专项规划，中心城区实现控详规划全覆盖，330个图层叠合的全域“一张蓝图”基本形成。深入实施存量土地攻坚，解决“净地不净”55宗、闲置土地67宗，盘活了一批停缓建工程，促进了土地资源节约集约利用。

　　加快重大基础设施建设。京沈高铁沈阳至承德段、新民北至通辽高铁同时开通。地铁2号线北延线投入运营，9号线试运行，10号线实现轨通，4号线建设有序推进。中央大街、东塔跨浑河桥、胜利大街阳光路立交桥、东一环快速路等路桥工程竣工通车。南北快速干道、浑南新城、南运河三条综合管廊工程竣工。大辛、西部、老虎冲生活垃圾焚烧发电项目同步建设，全市餐厨垃圾实现集中统一收运。

　　着力补齐城市运行短板。统筹推进老旧管网改造，完成供热管网改造76公里、燃气管网195公里、供水管网1346公里。实施排水防涝工程50余项，主城区汛期严重积水问题得到缓解。提高供电保障能力，11项电力工程投入运行。多措并举解决停车难问题，新增停车泊位20万个。实施重点拥堵路段潮汐车道改造，打通陵园北街等14条梗阻路、断头路。新开调整公交线路36条，新增更新纯电动公交车319台。

　　(六)全力以赴抓好三城联创。坚持全员发动、全线压上、全力而出，向积久积深积重积难的问题宣战，国家卫生城创建高分通过专家暗访和技术评估，国家食品安全示范城创建率先通过新国标省级验收。

　　城市面貌焕然一新。围绕“清、拆、管、建、疏、查、打、宣”八条主线，强化工程措施、管理措施、政策措施、改革措施，实施大巡查、推动大整改、促进大提升。改造农贸市场101个、公共厕所172座、背街小巷560条，完成裸露地面绿化覆盖300余万平方米，拆除违建950多万平方米，规范废品收购站1200余处，全面整治195个城中村及城乡接合部卫生环境，系统治理露天烧烤、占道经营等城市顽疾，不断加强公共卫生、病媒防制、疫病防控等工作，市民对创卫的满意率达到93.5%。

　　城市治理能力显著提升。实行周调度制度，建立现场指挥部、巡查督办、第三方暗访和专家评估、考核奖惩等工作机制。依托数字化城管平台，全面建立城市网格化管理体系，形成了问题发现、立案、派遣、结案、监管的工作闭环。加强全社会动员，推行河长、路长、楼长、场长、店长“五长制”，组织百万志愿者，基本实现一街路一组织、一社区一队伍。健全从农田到餐桌的全链条食品安全监管体系，有效保障了百姓舌尖上的安全。

　　民心士气昂扬向上。创城从知耻后勇的起点出发，走过了一段极不平凡的奋斗历程，全市上下都付出了巨大的辛苦，其意义远远超出了创城本身。广大干部群众所表现出来的那种众志成城的斗志、勇往直前的执着、无私奉献的品格，无时无刻不在彰显着向上向善的正能量、展现着奋勇争先的精气神、诠释着新时代的沈阳精神。各级干部在矛盾问题集中的创城实践中，磨砺了顽强意志，练就了过硬本领，锻造了务实作风，焕发了奋斗激情。这是我们推动新一轮振兴发展的宝贵财富！

　　(七)扎扎实实打好三大攻坚战。坚持守住底线、步步为营，不断筑牢决胜全面小康的坚实基础。

　　重大风险得到有效防控。化解政府性债务202亿元，超额完成年度任务。市属国企资产负债率整体降到66.6%。坚持市场化法治化原则，稳妥处置重点企业债务风险、涉众型非法集资风险，对存在股权质押风险的上市公司实施了救助。完善精准调控措施，开展商品房预售资金监管，促进房地产市场健康发展。非洲猪瘟疫情得到有效控制。

　　精准脱贫成果持续巩固。坚持摘帽不摘政策、力度不减、力量不撤，建立精准扶贫管理系统，投入扶贫资金8.7亿元，优选1400余名干部驻村帮扶，巩固扶持已脱贫人口18176人。支持4400个建档立卡贫困户发展产业扶贫项目，将3496名贫困人口纳入低保。实施新农合、大病保险、医疗救助和特殊补偿等一揽子政策，大幅减轻了贫困人口看病负担。

　　污染防治取得扎实成效。坚持科学治霾、精准治霾、协同治霾、铁腕治霾，综合实施燃煤锅炉拆除改造、散煤替代、扬尘管控、秸秆综合利用和散乱污企业治理，空气质量优良天数达到285天。全面推行五级河长制，出台全国首个黑臭水体管理规定，新建改造17座污水处理厂，环城水系截污工程投入使用，河流断面水质总体稳定。完成水土流失治理17万亩，祝家积存污泥加快处置，大辛、老虎冲积存渗沥液达标排放。

　　(八)持续加强保障改善民生。坚持抓好基本民生、保障底线民生、解决热点民生，群众获得感不断增强。

　　就业和社会保障进一步加强。新增城镇就业11.9万人，增长22.6%，城镇登记失业率3.1%。开展失业保险“援企稳岗”，惠及企业6700余户。养老金按时足额发放，城乡低保标准、医保政府补助标准持续提高。积极推进退役安置，积极解决历史遗留问题，退役军人服务管理工作得到加强。实施医疗救助14.4万人次、群众急难救助1.1万人次，孤儿、困境儿童、留守儿童、失独家庭等困难群体得到妥善照顾。

　　重点民生工程扎实推进。开通住房租赁交易平台，新增租赁住房3.7万套。改造棚户区1.66万套、老旧小区151个、农村危房391户，解决了2300户居民超期回迁和1.7万个家庭办证难问题。全市198个养老机构实现医养结合，二级以上综合医院全部开设老年病科(门诊)，新增区域性居家养老服务中心12个。新建改造6.86万农村居民安全饮水设施，全市学校和托幼机构实现“明厨亮灶”。在公共机构和350个小区开展生活垃圾分类试点。新增清洁供暖500万平方米。

　　社会事业全面进步。提高学前教育奖补标准，新增公办幼儿园学位1200个，义务教育超大班额全部消除，“择校热”问题有效解决，中德学院、宝马实训中心等双元培育基地开工建设。公立医院综合改革成效明显，基层医疗卫生机构实现医联体全覆盖。市博物馆、帅府红楼群展区等文化项目开工，中国锡伯族博物馆开馆，中国沈阳合唱节、国际钢琴节等文化活动蓬勃开展，评剧《黄显声》等文艺创作精彩纷呈。举办沈阳国际马拉松、“和平杯”国际青少年足球赛等赛事活动，我市荣获第十三届省运会金牌、奖牌、总分三项第一。沈阳通过全国文明城市测评复检。

　　社会治理能力不断提高。开展社区服务模式创新试点，新建社区服务站(中心)12个。法库县成为全国社会治理创新示范县。深入推进扫黑除恶专项斗争，累计打掉涉黑组织4个、涉恶集团23个、涉恶团伙98个，破获刑事案件337起。扎实开展信访矛盾减存控增攻坚行动，积案化解率达到63%，新案同比下降49%。实施安全生产“三项行动”，推进全市视频监控“雪亮工程”，提升社会治安综合防控能力，有效维护了社会稳定大局，增强了人民群众的安全感。

　　一年来，我们自觉接受人大及其常委会法律监督、工作监督和政协民主监督，提请市人大常委会审议法规草案4件，发布政府规章7件，754件代表建议、425件政协提案全部办复，切实将代表、委员的真知灼见转化为推动振兴发展的实际举措。双拥共建、国防动员、人防和民兵预备役建设持续加强。国家安全、保密、审计、仲裁、民族宗教、贸促、侨务、邮政、广播电视、新闻出版、气象、防震减灾、公积金、档案、修志、文史、残疾人、红十字会等各项事业都取得了新成绩。

　　各位代表，过去一年是不平凡的一年。宏观经济形势严峻复杂，风险挑战明显增多，改革发展任务异常繁重。面对诸多考验，我们坚持旗帜鲜明讲政治，牢固树立“四个意识”，不断强化“四个自信”，坚决做到“两个维护”，扎实开展中央巡视反馈意见整改，彻底肃清薄熙来、王珉等人流毒和恶劣影响，持续净化修复政治生态。我们积极践行新发展理念，高标准推进中央生态环保督察、国务院大督查等反馈意见整改落实，深入开展解放思想推动高质量发展大讨论，进一步理清发展思路，明确发展目标，细化发展举措。我们注重工作方式方法创新，坚持目标导向，强化过程控制和流程再造，推行挂图作战，横向上统筹协调，纵向上以上率下，一竿子插到底，不断提升政府工作体系能力。我们切实转变工作作风，积极走进企业、走进项目、走进基层、走进群众，坚持不为不办找理由、只为办好想办法，重实干、强执行、抓落实。特别是习近平总书记视察辽宁，主持召开深入推进东北振兴座谈会并发表重要讲话，进一步激发了全社会聚力振兴的强大精神力量，人心士气更加高涨，干事创业氛围更加浓厚！

　　各位代表，过去一年成绩的取得，得益于党中央、国务院的亲切关怀，得益于省委、省政府和市委的正确领导，得益于市人大及其常委会、市政协的有力监督和支持，是全市人民共同奋斗的结果。在此，我谨代表市政府，向全市人民，向人大代表、政协委员、各民主党派、各人民团体、社会各界人士以及离退休老同志，向驻沈人民解放军、武警部队官兵、消防救援队伍和中省直单位，向关心支持沈阳振兴发展的港澳台同胞、海外侨胞和国际友人，致以崇高的敬意和衷心的感谢！

　　各位代表，去年我市地区生产总值未能实现预期目标，有金融周期、经济周期、房地产周期相互叠加的影响，有结构性矛盾、体制机制性障碍尚未实现根本突破的原因，也反映出我们工作中还有许多短板和问题，主要是：营商环境仍有差距，一些政策措施落实不到位，企业获得要素成本较高，市场化服务体系不健全，诚信政府、法治政府建设还需加强。发展新动能不足，创新能力还不够强，新增长点不够多，一些企业经营困难，新经济、县域经济、民营经济发展不充分。对外开放步伐不大，投资自由化、贸易便利化等制度创新突破不够，开放平台和通道带动作用不强，利用外资和对外投资规模偏小，服务贸易发展明显滞后。城市治理比较粗放，规划管控体系还不完善，城市功能还有许多欠账，水体污染、交通拥堵、停车难等问题尚未有效解决，教育、医疗、养老等社会事业还有许多短板。思想观念解放不够，一些干部仍然存在不切实际、墨守成规、急于求成、坐等靠要的思想，不想为、不敢为、不善为都有所表现。对这些问题，我们将下大力气予以解决。

　　二、2019年重点工作安排

　　今年，是新中国成立70周年，是决胜全面建成小康社会第一个百年奋斗目标的关键之年。做好政府工作意义重大，总的要求是：以习近平新时代中国特色社会主义思想为指导，深入贯彻习近平总书记在辽宁考察时和在深入推进东北振兴座谈会上的重要讲话精神，根据中央及省委、市委经济工作会议部署，认真落实“五个坚持”要求，着力补齐“四个短板”、奋力推进“六项重点工作”，继续打好三大攻坚战，以深入解放思想为先导，以优化营商环境为基础，实施开放创新双轮驱动，激发市场主体活力，加快产业转型升级，统筹抓好稳增长、促改革、调结构、惠民生、防风险、保稳定等工作，全力保持经济持续健康发展和社会大局稳定，开创沈阳高质量发展、新时代振兴的新局面。

　　今年经济社会发展主要预期目标是：地区生产总值增长6.5%左右，一般公共预算收入增长7%，固定资产投资增长10%，社会消费品零售总额增长7%，城乡居民收入增长与经济增长同步；全社会研发投入稳步提高；单位地区生产总值能耗、水耗以及主要污染物排放持续下降；各类风险得到有效防控。

　　各位代表，完成今年目标任务，必须解放思想、大胆改革创新，不断强化自我革命的思想自觉、行动自觉，敢于打破利益固化的藩篱，敢于突破体制机制的积弊，敢闯敢试、敢为天下先，唱响改革创新的时代最强音。必须增强危机感、紧迫感，沈阳仍处在滚石上山、爬坡过坎的关键阶段，不进则退、慢进也是退，要以时不我待、只争朝夕的奋斗姿态履行新使命、施展新作为，快一点、再快一点。必须坚持目标导向、问题切入，紧紧围绕建设国家中心城市、实现“万亿千亿”目标，扬长避短、扬长克短、扬长补短，着力解决发展不平衡不充分的问题，不断提升区域竞争力。必须保持战略定力、坚定发展信心，坚持发展第一要务，全面落实高质量发展的指标体系、标准体系、政策体系和考核体系，深入推进供给侧结构性改革，多做打基础、利长远的事情。必须践行以人民为中心的发展思想，始终把群众的安危冷暖记在心上，把工作触角向群众的身边延伸，着力解决群众所急所需所忧所盼，着力攻难点、治痛点、疏堵点，不断增强人民群众的获得感、幸福感和安全感。

　　今年，重点做好以下几方面工作：

　　(一)努力打造营商环境一流城市。坚持持续发力、久久为功，推动营商环境建设再上新台阶。

　　强化突出问题整治。围绕企业和群众办事多次跑、跑多门、政务服务前置手续多等问题深化办事难专项整治，积极开展政府和企业失信、跨区拉税源、市场准入门槛高等专项整治。突出抓好政府失信问题整治，建立欠账清单，制定偿还计划，两年内解决拖欠民营企业工程款、招商引资政策未兑现问题，以实际行动取信于企、取信于民。对标营商环境建设标准，继续开展“企业评、群众议”，努力营造人人是环境、处处是环境、时时是环境的浓厚氛围。

　　优化金融生态环境。鼓励引导金融机构服务实体经济，推动城商行、农商行业务逐步回归本源。重构政策性担保体系，创新银企保对接模式。加快多层次资本市场建设，发挥区域股权市场培育功能，完善梯度培育机制，支持企业上市。完善基金体系，用好产业引导基金，设立直投基金，大力发展私募股权基金，推进一批基金小镇建设。建立中小微企业综合金融服务平台和企业征信平台，着力解决信息不对称、信用不充分问题。守住不发生区域性、系统性金融风险的底线。

　　加快建设人才高地。树立大人才观，不拘一格引进培养各类优秀人才，壮大创新型企业家队伍、科技人才队伍、技能人才队伍和农村实用人才队伍。优化人才政策，完善认定标准，细化工作流程，及时跟进调整，切实提高人才政策实效性、精准性和可操作性。加强人才服务，在安家落户、子女教育、医疗养老等方面为人才提供更多便利，增设人才交流工作站，推进人才驿站、人才公寓建设，让各类人才在沈阳工作有机会、创业有平台、发展有空间。

　　(二)全面深化重点领域关键环节改革。聚焦突破体制机制障碍，持续推进全面创新改革走深走实，坚定不移将各项改革进行到底。

　　加大国资国企改革力度。以混合所有制改革为突破口，积极引入战略投资者，推进集团层面混改，加快二三级公司混改。完善法人治理结构，加强国有企业党的建设，全面落实外部董事制度，扩大董事会市场化选聘经理层试点。探索管资本有效途径，完善对国有资本投资运营公司的授权经营体制，不断增强国有资本流动性。强化战略管控和风险防控，建立资产负债约束制度和风险预警机制，推行外派总会计师制度。巩固“三供一业”分离移交成果，开展“僵尸企业”处置“回头看”，基本完成厂办大集体改革，两年内完成国企退休人员社会化管理。

　　深化放管服改革。开展政务服务事项标准化体系建设，进一步统一前置要件、办事流程、数据归集等标准。大力实施“一网一门一次”改革，运行一体化在线政务服务平台，政务服务事项“网上办”不低于90%，试点推进便民服务事项“全区通办、全市通办”。完善商事主体综合服务平台功能，进一步压减企业开办、注销时限。深入推进工程建设项目审批制度改革试点，项目审批时限再压缩30%以上。积极推行“双随机、一公开”监管、大数据监管等新型监管方式，提高事中事后监管水平。

　　深化财政体制改革。推进全口径预算管理，开展财政支出绩效监控和绩效评价，提高财政资金使用效率。严格防控政府债务风险，落实债务化解三年行动计划，强化隐性债务管控，做好地方政府债券发行工作，有效防范基层财政运行风险。完善县乡财政管理体制，推进开发区独立预决算管理创新，增强自我发展能力。树立过紧日子的思想，一般性支出压减5%以上。

　　推进开发区高质量发展。向开发区下放建设项目环评、内资有限公司登记等审批权限。建立健全开发区产业空间规划、控详规划，明晰产业定位，大力推行“开发区+主题园区”模式。推动各开发区平台公司实质运营，完善开发区配套功能，加快教育、医疗等服务资源布局。加大招商引资力度，国家级开发区至少引进一个50亿元以上产业项目，省级开发区至少引进一个20亿元以上产业项目。

　　(三)着力做实做强做优实体经济。提升支柱产业、壮大新兴产业、培育特色产业、填补空白产业，加快产业转型升级，争创制造业高质量发展示范区。

　　突出抓好项目建设。推行领导包保、项目管家和现场指挥部等工作机制，全年推进亿元以上项目不少于1300个，6月底前续建项目100%复工，9月底前新建项目100%开工。坚持把“双招双引”作为各级政府“一号工程”，围绕培育壮大千亿产业集群，压实招商责任，完善考核办法，强化以商招商、中介招商，深耕欧美、日韩等国家地区以及京津冀、长三角、珠三角等重点区域，在引进重大项目上实现新突破，全年利用外资、引进内资增长15%以上。

　　加快传统制造业升级。依托国家重大专项，提高重大技术装备首台套研发、设计、制造及系统集成能力。围绕建设世界级汽车制造中心，抓好华晨宝马新工厂、华晨雷诺等重大项目建设，积极引进高端品牌、高端车系，提升汽车产业链层级。全面实施“电动沈阳”计划，培育新能源汽车产业增长引擎。实施新一轮企业技术改造升级工程，新增5家智能工厂、10家数字化车间，组织实施200个技改项目。鼓励企业管理创新，引导更多企业向精益管理要效益。

　　大力培育新产业新模式。以机器人未来城、国家机器人创新中心等重大项目、重大平台为引领，积极构建机器人产业生态圈。推动IC装备与零部件核心企业扩能升级，积极抢占人工智能领域制高点。加快发展航空产业，提高民用航空配套能力，支持通航龙头企业做大做强。推进北方生物医药谷、眼产业基地等重大项目建设，做大生物医药、数字医疗产业集群。突出抓好产业互联网建设，大力培育平台型企业，大规模推进5G网络基础设施建设，大范围展开5G网络产业示范应用。全面实施未来产业发展规划，向智能制造系统、新材料、区块链等前沿领域抢滩布局。

　　推进军民深度融合发展。聚焦航空、新材料等重点领域，加快军民融合“三园区、一基地”建设，推动中航发燃机公司总部落地，抓好沈飞A220系列新工厂、无人机生产基地等重点项目实施，加快推动东塔机场搬迁。以军工国企混改为契机，引导地方企业融入军工产业链。组建军民融合产业基金，搭建军民融合企业、项目、创新服务平台，设立军民融合技术交易中心，争创国家军民融合创新示范区。

　　加快发展现代服务业。大力发展全域旅游，促进文旅融合，整合资源打造精品旅游线路。推进盛京古城等旅游项目建设，推动北市场创建国家4A级旅游景区，力争盛京皇城创成国家5A级旅游景区。全面活跃会展经济，办好制博会、国际机器人大会、国际飞行大会、宝马供应商大会、华为用户大会等大型会展活动。深化国家现代物流创新发展试点城市建设，有序推进国家供应链体系建设试点任务，搭建现代物流公共信息服务平台，举办2019年全国物流园区工作年会。大力培育科技服务、工业设计、工业软件等生产性服务业，发展特色化、专业化楼宇经济。加快发展电子商务，促进五爱、南塔等市场转型升级，鼓励发展新型连锁便民超市。实施太原街等商圈改造，推动中街创建高品位步行街，积极发展夜间经济。

　　(四)充分释放全社会创新创业活力。围绕建设东北亚科创中心目标，完善创新体系，提升创新效能，夯实创新沈阳建设基础。

　　提高自主创新能力。深化院地合作，整合创新资源，围绕机器人、IC装备、人工智能等13个重点领域，实施百项“卡脖子”技术联合攻关。聚焦关键共性技术、前沿引领技术、现代工程技术、颠覆性技术研发与转化，持续推进“双百工程”。以自主创新示范区为载体，加速创新资源集聚，推进材料科学国家研究中心等重大创新平台建设，发挥平台溢出效应，建设“先进材料+智能制造”国家创新高地。

　　加速培育创新主体。进一步完善“科技型中小企业-高新技术企业-科技小巨人企业-瞪羚企业”梯度培育体系，科技型中小企业达到3000家，高新技术企业突破1500家。支持企业加大研发投入，建立研发机构，引进高端人才和创新团队。深入推进科技金融结合试点，遴选百家企业进入科技金融重点企业库，引导科技创新基金投向初创期、种子期企业，支持科技型企业登陆上海科创板。

　　提升科技成果转化水平。引导高校院所面向沈阳创新需求，建设科技成果转化基地。推进智慧医疗、增材制造等10个产业技术研究院建设，组建手术机器人、智能网联汽车等协同创新中心，新型研发机构达到20家。增强东北科技大市场、沈阳科技条件平台、技术转移示范机构专业化市场化服务能力，举办科技成果对接会等系列活动，全年技术合同成交额增长15%以上，本地成交额占比达到40%以上。

　　优化创新创业生态。推进锦联双创基地、三好双创大街、国际软件园等重点创新创业基地建设，培育壮大专业化众创空间、科技企业孵化器和加速器，全市双创孵化载体面积达到500万平方米，集聚创新创业人员达到15万人以上。加快中国(沈阳)知识产权保护中心建设，启动中德产业园知识产权仲裁院建设。着力打造双创升级版，积极开展全国“双创周”系列活动，培育“盛菁汇”等沈阳双创品牌，吸引创业项目、集聚创业英才，大力培育新时代创业者、新生代企业家。

　　(五)不断拓展对外开放的深度广度。坚持引进来和走出去并重，提升开放能级和层次，加快建设开放合作新高地。

　　深度融入共建“一带一路”。积极参与创建辽宁“一带一路”综合试验区和中国—中东欧“16+1”经贸合作示范区，筹办第五次中国—中东欧国家地方领导人会议，举办中俄高新技术工业展览会，推进中俄经贸物流东方快车等合作项目，打造“一带一路”东北亚枢纽城市。扩大国际产能合作，支持企业海外并购，发展境外产业园区。积极融入中蒙俄经济走廊和中日韩经济合作，深化与德英法等国家合资合作，全面提升我市在经济、科技、文化、教育、卫健、青年等领域的开放合作交流水平。

　　加强平台通道建设。推进自贸区提速发展，紧紧抓住制度创新，全面完成104项国家复制推广任务和115项省总体方案任务，聚焦东北亚开放合作等重点领域大胆闯、大胆试、自主改，努力形成可在全国复制推广的制度创新经验。抓好综保区桃仙园区封关运行，建立统筹联动通关模式，积极培育跨境电商、维修再制造等服务贸易形态，力争实际利用外资、进出口总额均实现倍增。推进中德产业园高水平建设，全面对接德国工业4.0，加快国际社区、国际学校规划建设，争取100个项目签约落地。推动中欧班列常态化运营，提高沈阳本地货源比例。进一步加密日韩等国际航线。推动沈阳港合资公司组建，加快核心港区建设，实现陆港空港联动发展，争创国家临空经济示范区。

　　推进外贸转型升级。加强出口基地建设，组建国有贸易公司，积极开拓欧洲、日韩、东南亚等重点市场，扩大机电产品、高新技术产品、农产品等优势产品出口。完善进口口岸功能，扩大高端装备、优质消费品等产品进口。抓好国家服务外包示范城市建设，大力发展数字贸易、文化贸易等新兴业态。加快跨境电商综合试验区建设，推进国际物流产业基地等项目落地实施，打造3个跨境电商特色园区。

　　深化区域交流合作。编制实施沈阳经济区一体化战略规划，构建发展协调、利益分享和创新合作机制，完善交通网络、产业分工、协同创新和公共服务体系，不断提高连接聚合扩散能力。主动承接北京疏解非首都功能，实施京沈IC装备产业联盟等一批合作项目，推动中关村科技成果加速在沈转化。加强与江苏省各市合作，在发展民营经济、科技创新、企业协作配套等方面取得新成效。积极与长江经济带、粤港澳大湾区重点城市开展对接，复制先进地区改革创新举措，实施一批跨区域合作项目。

　　(六)毫不动摇支持民营经济发展壮大。坚持同规则、同待遇、降门槛，加大对民营企业发展的扶持力度。

　　切实减轻企业负担。降低企业税费成本，确保国家更大规模减税降费政策落地落实。降低物流成本，扩大物流智能终端覆盖，解决配送“最后一百米”问题。降低用地成本，对优先发展产业项目在用地价格、供地方式等方面落实扶持政策。降低用能成本，扩大直购电规模，下调用水工程取费比例，对诚信企业实行免收或减半收取用气预付费政策。降低用工成本，继续实施阶段性降低失业保险费率政策。降低配套成本，提高产业集群配套率，促进大中小企业融通发展。

　　着力缓解融资难融资贵问题。将应急转贷资金规模扩大到10亿元以上，鼓励民营资本开展应急转贷业务，满足更多中小企业融资需求。扩大助保贷专项资金规模，完善风险补偿机制，积极组织合作银行向中小微企业开展助保金贷款业务。扩大应收账款质押融资贷款试点，引导保险资金开展融保通业务。设立100亿元纾困基金，纾解企业流动性风险。

　　营造公平竞争的市场环境。清理民间投资审批事项，实施市场准入负面清单制度，组织涉及民营经济的知识产权保护、反不正当竞争行为等政府规章的立改废释。大力实施PPP项目，引导社会资本进入基础设施、资源环境、公共服务等领域。鼓励民营企业参与国企改革。搭建绿色通道、予以持续支持，大力培育“专精特新”中小企业和民营经济“领头羊”，争取实现“个转企”2000户、“小升规”200户、“规升巨”20户。

　　加快构建亲清政商关系。健全重大经济政策制定企业家参与机制，建立各级政府联系商会协会制度。坚持“有求必应、无事不扰”，深入开展“万人进万企”主题活动，竭力为民营企业排忧解难。建立健全产需对接、科技成果对接、人才培训等服务平台，为中小微企业提供更加全面、精准的服务。开展民营企业就业百强、纳税百强、创新百强、规模百强评选表彰，讲好民营企业创业故事，大力弘扬企业家精神。

　　(七)坚定不移推进乡村振兴战略。坚持农业农村优先发展，激活农村要素，改善农村环境，补齐县域经济短板，让农业强起来、农村美起来、农民富起来。

　　大力发展都市农业。坚持绿色高效导向，建设高标准农田35万亩、设施农业3000亩，农业综合机械化率提高到86%。建设百万亩蔬菜基地，发展一村一品、一乡一业，持续培育沈阳大米、寒富苹果等农业品牌。不断提高农产品精深加工比例，加快建设“生产+加工+科技”、一二三产融合发展的现代农业产业园区。开发乡村旅游精品线路，实现乡村旅游成线成网，打造10个乡村旅游示范村，培育宜业宜居宜游的特色小城镇。积极发展“互联网+农业”，推广农村电商“法库模式”，农村电商主体突破1000家。

　　不断深化农村改革。落实农民对集体经济收益的分配权，推动资源变资产、资金变股金、农民变股民，完成60个村集体产权制度改革试点。稳妥推进集体经营性建设用地入市，发展乡村旅游等新业态。继续扩大承包地流转，培育新型经营主体300家。探索宅基地使用权流转，激活闲置资源。积极发展农村普惠金融，加快农信社改制，设立农业产业基金，合理引导工商资本下乡。

　　加快改善农村人居环境。坚持规划先行，分类梯次推进，全面实施农村人居环境整治三年行动。有序推进农村生活垃圾和污水处理，新建改造21座乡镇垃圾中转站，完善3个乡镇、18个村庄污水处理设施，开展村屯河道整治，新建改造12.5万人饮水安全设施。扎实推进农村厕所革命。完善农村生态补偿机制，调动农民退耕还绿积极性。保护好历史文化名村，实施224个村庄美化亮化绿化，新建改造农村公路1000公里、村内道路1000公里，建设10个美丽宜居示范村。

　　提高乡村治理水平。坚持自治、法治、德治相结合，完善村党组织领导的充满活力的乡村治理机制。支持种植能手、能工巧匠、返乡青年等乡土人才创新创业，带动农民致富、技艺传承、农业发展。开展文明村镇创建活动，弘扬邻里守望、重义守信、孝老爱亲等传统风尚，共建文明乡村、善治乡村。

　　大力发展飞地经济。围绕强县富民，实施三年行动计划，完善政策体系，构建利益分享机制，建立科学合理的指标核算制度。做优做强主导产业清晰的县域工业园区，拓宽基础设施融资渠道，鼓励标准化厂房建设，提高对产业项目的承载力。构建全员招商体系，强化乡镇招商职能，每个乡镇至少引进1个投资3000万元以上工业项目、1个2000万元以上农产品深加工项目。

　　(八)加快提升中心城市功能品质。深化城市“治改提创”，巩固创城成果，不断完善城市功能，促进城市内涵发展。

　　提高规划管控能力。编制新一轮国土空间规划，完善供热、燃气、电力、污水、养老设施、地下空间等专项规划，推动产城融合、职居平衡、功能均衡。深化“多规合一”，厘清保护和开发边界，明晰各类用地布局，切实发挥规划的引领管控作用。做好第三次国土调查工作，强化存量土地攻坚，加大力度盘活各类闲置低效资源。

　　完善综合交通体系。启动沈白高铁建设，提升沈阳北站功能，扩大南站综合运输能力。积极推动桃仙机场二跑道项目建设。加快地铁骨干线网建设，9号线通车运营，10号线试运行，2号线南延线和3号线同时开工。推进城市主次干道连接，完成昆山西路与304国道连通高架桥以及长安桥、云龙湖桥接线路工程。继续完善快速交通系统，完成长青街、胜利南大街、沈辽路快速路工程，实施浑南大道快速路、沈康高速连接线建设工程。

　　保障城市运行安全。统筹地上与地下、兼顾新城与老城，新建70公里、改造185公里燃气管网，改造300公里供水内网、40公里供热管网。实施盛京500千伏枢纽工程和滂江等8处220千伏区域电源点建设。完成大伙房输水配套西部净水厂二期工程，推进辽西北供水配套工程。继续开展排水防涝工程，新建改造肇工街等7大排水系统、龙王庙等7座泵站，对54处积水点进行排涝除险，对浑河城市段进行堤防加固。着力解决停车难、行车难问题，新建盘活15万个停车泊位，打通12处梗阻路段，启动8处铁路道口平改立工程，新建15处行人过街设施。新开调整公交线路20条，新增更新公交车380台，进一步提高公交分担率。

　　完善创城长效机制。坚持创城工作标准不放松，坚持每周调度、巡查督办、第三方暗访评估等工作机制。不断深化网格化管理，进一步落实“五长制”，持续巩固市容环境整治成果。深化环卫扫保市场化改革，推动基础设施、市政公用等领域政事分开、建管分离。常态化开展志愿者服务活动，引导市民自我管理、自我服务，共建共治共享良好人居环境。

　　提升城市文化魅力。加强城市设计，做好城市街道、建筑和城市色彩控制引导，彰显地域文化特色。物化活化历史文化资源，加强重点历史建筑保护修缮，充分利用老旧厂房拓展功能空间，积极推动具备条件的历史遗迹复原复建，抓好盛京皇城改造、北大营抗战陈列馆建设等重点文化项目。推动老龙口酒文化一条街等老字号街区建设，打造集文化遗产传承、辽沈风貌展示、品牌文化推广于一体的“城市文化客厅”，加快建设文化名城。

　　(九)持之以恒推进生态文明建设。认真落实中央生态环保督察整改任务，推进生态环境整体保护、系统修复和综合治理，努力建设美丽沈阳。

　　强化污染治理。着力“巩固气、突破水、治理土”。努力改善空气质量，持续整治“煤车尘炉”和挥发性有机物，争取空气质量优良天数比率再提高1-2个百分点。加快水体治理进度，推进辽河、浑河、蒲河、细河、北沙河等河流超标断面整治。推进东部、南部、白塔堡等污水厂新建扩建工程，抓好雨污混接整改工程。加强土壤治理修复，确保祝家积存污泥全面解决，大辛、老虎冲积存渗沥液处理按期完成。扩大生活垃圾分类试点，推进建筑垃圾资源化利用，提高危废处理能力，确保大辛、西部、老虎冲生活垃圾焚烧发电项目投入运行。

　　加快生态修复。建立健全自然资源管理体系，开展自然资源确权登记。强化森林、草原、湿地等生态系统保护，加强自然保护区管理，推进卧龙湖生态区和辽中蒲河、法库獾子洞、康平辽河国家湿地公园建设。提升辽河、浑河等重点河流生态功能，实施两岸生态封育、河口湿地建设和拦蓄水工程。大力实施黑土地保护战略，落实轮作休耕制度，促进化肥农药减量增效，开展沙化土地治理修复。完善“三环、三带、四楔、南北绿廊”绿地系统，实施人工造林15万亩。

　　发展绿色经济。深化循环经济试点，加强节能减排。积极推行绿色生产生活方式，开展农业节水行动，推进绿色工厂建设，推广装配式建筑和精装修，提高清洁供暖比例。积极发展节能环保、清洁能源产业，促进产业结构变“轻”、产业形态变“绿”。大力发展冰雪经济，制定实施冰雪产业发展规划，推进滑雪滑冰场馆设施改造建设，积极开发冰雪旅游项目，办好国际冰雪节、百万市民上冰雪等文化旅游活动。

　　(十)推动发展成果更多更公平惠及民生。确保民生投入只增不减，切实解决好社会关切，不断提升群众幸福指数。

　　着力促进充分就业。实施积极的就业政策，以创业带动就业，抓好高校毕业生、农民工、退役军人等重点群体就业服务，城镇新增就业10万人，争取吸引12万高校毕业生就业创业。实施就业困难群体精准帮扶工程，帮助1万名就业困难人员实现就业。城镇登记失业率控制在3.6%以内。

　　着力兜牢民生底线。实施全民参保计划，加强社保基金监管，确保养老金按时足额发放。持续提高城乡低保、特困人员供养、优抚对象抚恤和医保政府补助标准，将孤儿、农村留守儿童、困境儿童关爱保障工作纳入绩效考核，完善社会救助体系。围绕“两不愁、三保障”，加大精准扶贫资金投入、力量投入，大力推进产业扶贫、教育扶贫、健康扶贫，开展资产收益扶贫试点，扩大重大疾病保障病种范围，做好扶贫与低保政策衔接，不断巩固脱贫成果。

　　着力改善养老服务质量。加强居家养老服务体系建设，新增区域性居家养老服务中心100个，改造社区养老服务站300个，打造城市15分钟养老服务圈，扎实推进农村居家养老服务试点。实施养老机构服务提升工程，扩大医养结合服务，发展养老护理服务、文化服务，满足居家老年人多层次、多样化的养老服务需求。进一步放开养老服务市场，鼓励民营资本参与公办养老机构改革，支持社会力量运营养老服务设施。

　　着力提升住房保障水平。坚持“房子是用来住的、不是用来炒的”定位，完善调控长效机制，强化精准施策，促进房地产市场平稳健康发展。加大公共租赁住房保障力度。持续改善群众居住条件，改造棚户区1.6万套、农村危房631户、老旧小区100个，治理老旧小区用电安全隐患，启动老旧小区加装电梯试点，加速解决产权证办理难、燃气拖期开栓、回迁安置超期等遗留问题。

　　着力办好人民满意教育。加快构建德智体美劳全面培养的教育体系。普惠性幼儿园覆盖率达到75%，实施农村教育质量提升计划，促进城乡义务教育均衡发展。适应新高考改革，深化中考制度改革。促进产教融合，抓好双元制职业教育基地建设。力争消除大班额，巩固“择校热”治理成果，做好弹性离校、课后服务工作，加大“乱补课”治理力度，切实维护校园及周边安全。全面落实国家综合防控儿童青少年近视实施方案，坚决遏制近视低龄化、重度化趋势，呵护好孩子们的身心健康。

　　着力建设健康城市。推进医疗卫生由以治病为中心向以健康为中心转变，深化为民惠民医改，扩大分级诊疗和家庭医生签约服务，稳妥开展医保药品集中采购和使用试点，确保群众用上质优价廉的药品。支持中医药事业发展，优化社会办医环境。完善群众性体育设施，推动足球国家青训中心建设，举办全国田径锦标赛暨国际邀请赛、沈阳国际马拉松、“和平杯”国际青少年足球赛等赛事活动，加快建设体育名城。

　　着力繁荣文化事业。用好用活沈阳优秀的历史文化、工业文化、红色文化资源，鼓励话剧《国徽》等文艺精品创作，培育盛京大剧院等院线品牌。发挥驻沈高校、文化团体作用，推进文创产品开发和文创产业集聚发展。依托“一河两岸”积极开展文化、旅游、体育等主题活动，举办2019沈阳合唱音乐季，建设125个村文化广场，组织实施艺术惠民演出、流动文化馆等文化惠民工程。

　　着力加强和创新社会治理。推动治理重心下沉，开展社区运行和服务机制创新试点，提高基层服务能力。坚持发展“枫桥经验”，发动和依靠群众就地化解矛盾。争创双拥模范城“九连冠”。推进“七五”普法，完善公共法律服务体系。保持扫黑除恶斗争高压态势，深挖彻查保护伞。加强市场监管，确保不发生区域性、系统性食品药品安全事故。强化安全生产管理，完善应急管理工作机制，防范遏制重特大安全事故发生。继续开展信访矛盾减存控增攻坚行动，确保社会大局和谐稳定。

　　各位代表，做好新一年工作，必须高举习近平新时代中国特色社会主义思想伟大旗帜，牢固树立“四个意识”，始终坚定“四个自信”，切实做到“两个维护”，继续重实干、强执行、抓落实，不断提高政府自身建设水平。

　　努力建设诚信政府。健全政府践诺守信奖惩机制，将政府履约、守诺情况等纳入绩效考核，坚决杜绝新官不理旧账，做到言必信、行必果，说到做到、办就办好，切实增强政府公信力和执行力，以政府诚信带动企业诚信、社会诚信，共建诚信沈阳。

　　努力建设法治政府。全面完成政府机构改革任务，落实“三定”方案，做到忠诚履职、尽责担当。依法接受人大法律监督和工作监督，自觉接受人民政协民主监督，主动接受社会和舆论监督，把议案、建议和提案办好办实。推动政府立法与时俱进，不断提高法治能力。

　　努力建设廉洁政府。全面加强政府系统党的建设，认真履行从严管党治党政治责任，严格落实八项规定精神，驰而不息纠正“四风”。推进改革限权、依法确权、全程控权，扩大政务公开，加强审计监督。始终把纪律和规矩挺在前面，干干净净做事，无愧于人民信任。

　　努力建设服务型政府。强化公仆意识，坚持问计于民、问需于民，确保政府决策顺民心、合民意。增强服务本领，提升服务效能，努力做到能为善为。坚持唯实务实求实，严细实快抓好工作落实，以实实在在的成果造福全市人民。

　　各位代表！开创沈阳高质量发展、新时代振兴的新局面，需要只争朝夕的劲头，需要久久为功的毅力，需要舍我其谁的担当。让我们更加紧密团结在以习近平同志为核心的党中央周围，在省委、省政府和市委坚强领导下，团结一心、迎难而上、拼搏实干，以优异成绩迎接中华人民共和国成立70周年！
